# Vitamin D-related genetic variants as predictive biomarkers for breast cancer in Jordanian women

**DOI:** 10.3389/fmed.2026.1816935

**Published:** 2026-05-28

**Authors:** Laith N. Al-Eitan, Maryam K. Alasmar, Mohammed S. Alorjani, Lina M. Sarhan, Sakher S. Sammour, Farah N. Aladwan, Hadil A. Al Refai, Sara S. Hasanat, Mira S. Quran, Mansour A. Alghamdi

**Affiliations:** 1 Department of Biotechnology and Genetic Engineering, Jordan University of Science and Technology, Irbid, Jordan; 2 Department of Pathology and Microbiology, Faculty of Medicine, Jordan University of Science and Technology, Irbid, Jordan; 3 Department of Anatomy, College of Medicine, King Khalid University, Abha, Saudi Arabia; 4 Genomics and Personalized Medicine Unit, The Center for Medical and Health Research, King Khalid University, Abha, Saudi Arabia

**Keywords:** breast cancer, CYP24A1, CYP27B1, CYP2R1, DBP, polymorphism

## Abstract

**Background:**

Vitamin D exerts a protective role in carcinogenesis by regulating signaling pathways implicated in tumor growth and progression. Variants in CYP2R1, CYP24A1, CYP27B1, and DBP have been reported to influence circulating 25(OH)D concentrations and provide evidence for a hypothesis that these polymorphisms may be associated with breast cancer risk by altering vitamin D metabolism and signaling. Therefore, the present study has been designed to assess the correlation of these gene polymorphisms with susceptibility to breast cancer.

**Methods:**

A case-control study was encompassed, including 300 patients with breast cancer and 300 healthy controls. Genotyping of CYP2R1, CYP27B1, CYP24A1, and DBP polymorphisms was determined employing PCR-based restriction fragment length polymorphism (RFLP) assays. Each of the polymorphisms was assessed for association with breast cancer susceptibility using multiple statistical approaches.

**Results:**

Significant associations were observed for CYP2R1 rs12794714 and CYP27B1 rs10877012, with the GG genotype of CYP2R1 and the TG genotype of CYP27B1 associated with a reduced risk of breast cancer. In contrast, no significant associations were observed for CYP2R1 rs10741657, CYP24A1 rs2248359, or DBP rs7041 and rs4588 variants.

**Conclusion:**

Our study suggests that certain genotypes in CYP2R1 and CYP27B1 might provide protective influences against the development of breast cancer in Jordanian females, although further studies are recommended to validate the results.

## Introduction

Vitamin D, which is also defined as 1,25-dihydroxyvitamin D3, is an essential steroid prohormone that is used to maintain musculoskeletal health in human body (1). Vitamin D has been implicated in numerous extraskeletal pathologies, with sufficient evidence supporting its link to infectious diseases (2), cardiovascular disease (3), autoimmune disorders (4), and cancer (5). Insufficient levels of vitamin D are linked to the development of multiple cancers, such as breast (6), colorectal (7), prostate (8), pancreatic (9), thyroid (10), hepatocellular (11), and ovarian cancers (12). Moreover, vitamin D supplementation has been shown to reduce cancer-related mortality by 16% (13).

Serum concentrations of vitamin D reflect interindividual variability and cannot entirely be accounted for by factors such as age, sun exposure, body mass index, or dietary intake (10). Evidence suggests that serum vitamin D concentrations are largely influenced by genetic inheritance (14). Several critical steps in vitamin D metabolism are influenced by genetic and epigenetic factors. Essential genes such as CYP2R1, VDR, CYP24A1, and CYP27B1 play a guiding role in this pathway, and their dysregulated expression has been linked to disturbance in vitamin D levels and cancer development (15–19). Studies utilizing Genome-wide association studies (GWAS) have demonstrated that single nucleotide polymorphisms (SNPs) in genes implicated in vitamin D metabolism are significantly associated with variations in 25-hydroxyvitamin D concentrations (1, 14).

Vitamin D refers to a group of fat-soluble compounds, including ergocalciferol, cholecalciferol, calcidiol, and calcitriol. Cholecalciferol is endogenously produced in the skin upon exposure to UVB radiation (290–320 nm) from 7-dehydrocholesterol, while ergocalciferol is obtained through dietary sources (20–22). The transport of both vitamin D isoforms in circulation occurs via their binding to vitamin D-binding protein (VDBP) (23). The activation of these isoforms involves two distinct hydroxylation processes. The initial hydroxylation takes place in the liver, catalyzed by the 25-hydroxylase enzyme (CYP2R1), leading to the formation of calcidiol [25(OH)D]. This metabolite stays in circulation for the longest period and is commonly measured to assess the levels of vitamin D in human serum (21). The second hydroxylation occurs in the kidneys and is catalyzed by the enzyme 1-alpha-hydroxylase (CYP27B1), which results in the synthesis of calcitriol [1,25(OH)D]. Calcitriol, the active biological form of vitamin D, anchors itself to the vitamin D receptor (VDR) on the membrane of target cells (24–26). After the vitamin D receptor (VDR) binds calcitriol, the complex is transported to the nucleus, where it forms a dimer with the retinoid X receptor (RXR), leading to the activation of vitamin D response elements (VDREs) as a transcription factor (25, 26). The inactivation of calcitriol and calcidiol achieved through successive hydroxylation reactions carried out by the CYP24A1 enzyme, which enhances the solubility of the compounds and facilitates their excretion via the kidneys (27, 28).

These VDREs facilitate the transcription of multiple genes involved in critical processes associated with cancer initiation and progression. These findings indicate that reduced serum calcidiol levels or impaired vitamin D function may contribute to carcinogenesis, potentially influenced by single-nucleotide polymorphisms (SNPs) in genes involved in vitamin D metabolism, including VDBP, VDR, CYP2R1, CYP27B1, and CYP24A1 (29–31). Previous studies have reported inconsistent or null associations between the majority of potential SNPs in vitamin D-related genes and breast cancer risk (32–36). Only a few studies have explored the relationship between cancer risk and SNPs in vitamin D-related genes beyond VDR (36–42). To our knowledge, few or no studies have specifically examined the role of CYP2R1, CYP27B1, CYP24A1, and DBP polymorphisms in breast cancer susceptibility.

Given the fact that cancer is a highly complex disease affected by numerous factors with intricate physiological regulation systems, an in-depth study of the possible direct or indirect contribution of genetic polymorphisms to the occurrence of cancer is warranted. Even though the relationship between genetic polymorphisms and cancer has been studied in various ethnicities in the context of breast cancer, insufficient data have been generated regarding the genetic factors responsible for the onset of breast cancer in the Jordanian Arab population. Thus, the necessity of carrying out further research in this gap area of knowledge signifies the importance of the current subject of research and aims to validate the relationship between the presence of polymorphisms in the genes CYP2R1, CYP27B1, CYP24A1, and DBP with the onset of breast cancer in the Jordanian Arab population.

## Materials and methods

### Participants

A total of 600 female participants from Jordan were enrolled in the study, comprising 300 breast cancer patients and 300 healthy individuals without a prior diagnosis of breast cancer who served as the control group. Participants were enrolled from the Chemotherapy Clinics of King Abdullah University Hospital (KAUH) and King Hussein Medical Center, in accordance with specific selection criteria. The study was designed and implemented in strict adherence to the highest ethical standards and received the Institutional Review Board (IRB) approval from Jordan University of Science with approval number (Apr2025/180-47). All participants signed written informed consent before being included.

Patients were enrolled based on predefined inclusion criteria, including a histopathologically confirmed breast cancer diagnosis, absence of HIV, HBV, or HCV infection and availability of clinical data in the KAUH patient registry. Excluded from the study were individuals who declined to provide written informed consent, those who had received a blood transfusion during surgery and those with incomplete clinical information. The control group consisted of individuals with no personal history of breast cancer or any other malignancy. Control participants were selected from the same ethnic background to reduce the risk of population stratification bias. Exclusion criteria for the control group included a prior diagnosis of cancer, a first-degree family history of breast cancer, chronic infections and recent blood transfusion. Individuals who declined to provide informed consent or biological samples were also excluded from the study.

Demographic data were collected for all participants, including age, body mass index (BMI), smoking status, age at menarche and family history of cancer. In addition, clinical characteristics of the patient group, such as menopausal status, estrogen receptor (ER) status, progesterone receptor (PR) status, clinical stage and other clinical variables, were recorded to evaluate their potential association with variability within the patient group.

### DNA extraction and genotyping

Purification of genomic DNA was utilized by employing the Puregene^®^ Blood Core Kit A (Qiagen, United States) in accordance with the manufacturer’s instructions from frozen samples of the peripheral blood collected in EDTA-contained tubes. DNA concentration and purity were determined with a NanoDrop spectrophotometer, and samples with an A260/A280 ratio of 1.8–2.0 were deemed suitable for downstream applications. The integrity of the DNA was further confirmed by electrophoresis on a 1% agarose gel. Extracted DNA samples were stored at −20 °C until further use.

PCR amplification of the target sequence of selected SNPs in the CYP2R1, CYP27B1, CYP24A1, and DBP genes was carried out using gene-specific primers. Each sample reaction contained template DNA, 10 μM of each forward and reverse primer, nuclease-free water and 2 × PCR master mix, prepared according to standard protocols. Amplified products were digested with 10 units of the specific restriction enzyme for each SNP, as presented in Table 1, for 2 h. The digested products were subsequently separated by electrophoresis on a 2% agarose gel and fragment sizes were determined using a 100 bp DNA ladder. The quality of genotyping was evaluated through re-genotyping of some of the samples that were randomly selected. Samples with unclear or ambiguous results were re-genotyping to ensure accurate genotype assignment. Detailed genotype data for all analyzed polymorphisms are provided in the Supplementary material.

**TABLE 1:** Primer amplicon design, restriction enzymes and resulting fragment sizes for selected SNPs.

Polymorphism	Primers	Restriction enzyme	Allele	Fragment size (bp)
CYP2R1	F: 5′-GGGAAGAGCAATGACATGGA-3′	MnII	A	256, 32
(rs10741657)	R: 5′-GCCCTGGAAGACTCATTTTG-3′		G	151, 105, 32
CYP2R1	F: 5′-GGAAGCTTTGGAGAGCTGAA-3′	*Fok*I	A	303
(rs12794714)	R: 5′-GCCATAAGTCCAACCAGGAA-3′		G	168, 148
CYP27B1	F: 5′-TTCAATTCCAGAACTTCAGAGC-3′	*Tfi*I	T	298
(rs10877012)	R: 5′-AACATAGTCGAACTGTCTCTAC-3′		G	195, 103
CYP24A1	F: 5′- AGTTAGGAAATGCGCCTTGAG-3′	*Sac*II	C	326
(rs2248359)	R: 5′- GGATCAGGTTGAAAGGATTCG-3′		T	226, 100
DBP (rs7041)	F: 5′-AAATAATGAGCAAATGAAAGAAGAC-3′ R: 5′-CAATAACAGCAAAGAAATGAGTAGA-3′	*Hae*III	T	483
			G	297, 186
DBP (rs4588)		*Sty*I	C	483
			A	305, 187

### Statistical analysis

Statistical analyses were employed using SPSS software, version 26.0 (SPSS Inc., Chicago, IL), along with the SNPstats web application available online at https://www.snpstats.net/start.htm. Hardy-Weinberg equilibrium (HWE) was assessed for each polymorphism, and allelic and genotypic frequencies were calculated. To establish the relationship between genetic variations and the risk of breast cancer, various models of inheritance were considered, and the results were expressed using odds ratios (ORs) and 95% confidence intervals (CIs). Genotype correlations to phenotype were assessed using Pearson’s chi-square test beside one-way analysis of variance (ANOVA). Haplotype analyses were performed to examine the combined effects of multiple SNPs within each gene studied on disease susceptibility. The criterion for statistical significance was set at *p* < 0.05. Multivariable binary logistic regression was performed to assess whether the investigated polymorphisms independently contribute to disease susceptibility, adjusting for potential confounders. A *post hoc* statistical power analysis was performed using G*Power software version 3.1.9.7, based on observed genotype frequencies between cases and controls, using a two-tailed test for differences between two independent groups and a significance level of 0.05. To correct for multiple testing, the effective number of polymorphisms was determined according to a previously reported method (43), and the significance threshold was adjusted using the Bonferroni correction (α/n, where α = 0.05 and *n* is the number of tests) (44).

## Results

### Baseline characteristics of the study population

The baseline demographic characteristics of the study population were comparable between breast cancer patients and controls. The mean age was 52.32 ± 11.39 years in patients and 52.35 ± 11.24 years in controls (*p* = 0.97). Similarly, no statistically significant differences were observed in BMI (28.86 ± 5.98 vs. 28.36 ± 5.92, *p* = 0.30), smoking status (14.2% vs. 16.6%, *p* = 0.44), or age at menarche (13.73 ± 1.65 vs. 13.82 ± 1.33, *p* = 0.52). In contrast, a family history of cancer was significantly more frequent among patients compared to controls (54.7% vs. 36%, *p* < 0.001).

### Minor allele frequencies and Hardy-Weinberg equilibrium (HWE) test

Minor allele frequencies (MAFs) of the genetic variants in the CYP2R1, CYP27B1, CYP24A1, and DBP genes were systematically examined in breast cancer patients and healthy controls to study associations of these variants with disease risk. To ensure reliability of the data, Hardy-Weinberg equilibrium was calculated for each polymorphism. Most variants were in HWE in both case and control groups, reflecting genetic stability and lack of confounding factors like selection or mutation. Unexpectedly, CYP27B1 SNP rs10877012 showed a significant deviation from HWE in the case group, as shown in Table 2.

**TABLE 2:** Hardy-Weinberg equilibrium status and minor allele frequency distribution of the target SNPs.

Gene	Control (*n* = 300)			Cases (*n* = 300)		
	MA	MAF	HWE *p*-value	MA	MAF	HWE *p*-value
CYP2R1 (rs12794714)	G	48%	0.73	G	43%	0.06
CYP2R1 (rs10741657)	A	27%	0.15	A	26%	0.88
CYP27B1 (rs10877012)	T	20%	0.1	T	20%	<0.0001
CYP24A1 (rs2248359)	C	43%	0.06	C	44%	0.19
DBP (rs7041)	T	46%	0.73	T	49%	1
DBP (rs4588)	A	13%	0.61	A	10%	0.18

MA, minor allele; MAF, minor allele frequency; HWE, Hardy-Weinberg equilibrium.

### Allele and genotype distributions of selected polymorphisms

Allele frequencies and genotype distributions for polymorphisms in CYP2R1, CYP27B1, CYP24A1, and DBP genes are presented in Table 3. Significant differences in genotype distribution were observed for rs12794714 in CYP2R1 (*P* = 0.04) and rs10877012 in CYP27B1 (*P* = 0.004), suggesting a potential association with breast cancer susceptibility. No significant correlations were revealed for the remaining polymorphisms. Following Bonferroni correction, only rs10877012 in CYP27B1 remained significantly associated with breast cancer risk.

**TABLE 3:** Allele frequencies and genotype distributions of targeted polymorphisms.

Gene	SNP_ID	Allele/genotype	Control (*n* = 300)	Cases (*n* = 300)	*P*-value
CYP2R1	rs12794714	A G	309 (52%) 291 (48%)	340 (57%) 260 (43%)	0.08
		A/A A/G G/G	81 (27%) 147 (49%) 72 (24%)	88 (29%) 164 (55%) 48 (16%)	0.04
CYP2R1	rs10741657	G A	435 (72%) 165 (28%)	444 (74%) 156 (26%)	0.55
		A/A A/G G/G	27 (9%) 108 (36%) 163 (55%)	21 (7%) 114 (38%) 165 (55%)	0.63
CYP27B1	rs10877012	G T	479 (80%) 121 (20%)	481 (80%) 119 (20%)	0.88
		T/T G/T G/G	17 (6%) 87 (29%) 196 (65%)	31 (10%) 57 (19%) 212 (71%)	0.004
CYP24A1	rs2248359	A C	343 (57%) 257 (43%)	333 (56%) 267 (44%)	0.56
		A/A A/C C/C	92 (31%) 159 (53%) 49 (16%)	84 (28%) 165 (55%) 51 (17%)	0.77
DBP	rs7041	G T	434 (73%) 162 (27%)	444 (74%) 156 (26%)	0.64
		G/G T/G T/T	89 (30%) 146 (49%) 65 (22%)	77 (26%) 151 (50%) 72 (24%)	0.46
DBP	rs4588	C A	522 (87%) 78 (13%)	539 (90%) 59 (10%)	0.08
		A/A C/A C/C	6 (2%) 66 (22%) 228 (76%)	5 (2%) 49 (16%) 245 (82%)	0.20

*P* < 0.05 was considered statistically significant. Following adjustment for multiple comparisons using the Bonferroni method (0.05/6), *P*-values < 0.0083 were considered statistically significant.

### Genetic model analysis

The association between polymorphisms in CYP2R1, CYP27B1, CYP24A1, and DBP genes and breast cancer risk was evaluated using multiple genetic models, with odds ratios (ORs) reported in Table 4. Significant associations were revealed for the CYP2R1 rs12794714 polymorphism under the codominant (OR = 0.61, *P* = 0.048) and recessive (OR = 0.60, *P* = 0.014) models. *Post hoc* power analysis indicated moderate power (68%) for this association. Additionally, the CYP27B1 rs10877012 polymorphism showed significant associations under the codominant (OR = 0.61, *P* = 0.004), recessive (OR = 1.92, *P* = 0.034) and overdominant (OR = 0.57, *P* = 0.004) models, with adequate statistical power (81%), supporting the reliability of this finding. No significant associations were observed for the remaining polymorphisms across the genetic models evaluated. After Bonferroni correction, only rs10877012 in CYP27B1 showed significant associations with breast cancer risk in the codominant and overdominant models (*P* < 0.008).

**TABLE 4:** Analyses of selected polymorphisms using different genetic models.

Polymorphism	Model	Genotype	Controls (%)	Case (%)	OR (95% CI)	*P*-value
CYP2R1 (rs12794714)	Codominant	A/A A/G G/G	81 (27%) 147 (49%) 72 (24%)	88 (29.3%) 164 (54.7%) 48 (16%)	1 1.03 (0.71–1.49) 0.61 (0.38–0.99)	0.048
	Dominant	A/A A/G-G/G	81 (27%) 219 (73%)	88 (29.3%) 212 (70.7%)	1 0.89 (0.62–1.27)	0.53
	Recessive	A/A-A/G G/G	228 (76%) 72 (24%)	252 (84%) 48 (16%)	1 0.60 (0.40–0.91)	0.014
	Overdominant	A/A-G/G A/G	153 (51%) 147 (49%)	136 (45.3%) 164 (54.7%)	1 1.26 (0.91–1.73)	0.16
CYP2R1 (rs10741657)	Codominant	G/G A/G A/A	163 (54.4%) 109 (36.3%) 28 (9.3%)	165 (55%) 114 (38%) 21 (7%)	1 1.03 (0.74–1.45) 0.77 (0.42–1.41)	0.57
	Dominant	G/G A/G-A/A	163 (54.3%) 137 (45.7%)	165 (55%) 135 (45%)	1 0.97 (0.71–1.34)	0.87
	Recessive	G/G-A/G A/A	272 (90.7%) 28 (9.3%)	279 (93%) 21 (7%)	1 0.73 (0.41–1.32)	0.3
	Overdominant	G/G-A/A A/G	191 (63.7%) 109 (36.3%)	186 (62%) 114 (38%)	1 1.07 (0.77–1.50)	0.67
CYP27B1 (rs10877012)	Codominant	G/G T/G T/T	196 (65.3%) 87 (29%) 17 (5.7%)	212 (70.7%) 57 (19%) 31 (10.3%)	1 0.61 (0.41–0.89) 1.69 (0.90–3.14)	0.004
	Dominant	G/G T/G-T/T	196 (65.3%) 104 (34.7%)	212 (70.7%) 88 (29.3%)	1 0.78 (0.55–1.10)	0.16
	Recessive	G/G-T/G T/T	283 (94.3%) 17 (5.7%)	269 (89.7%) 31 (10.3%)	1 1.92 (1.04–3.55)	0.034
	Overdominant	G/G-T/T T/G	213 (71%) 87 (29%)	243 (81%) 57 (19%)	1 0.57 (0.39–0.84)	0.004
CYP24A1 (rs2248359)	Codominant	A/A A/C C/C	92 (30.7%) 159 (53%) 49 (16.3%)	84 (28%) 165 (55%) 51 (17%)	1 1.14 (0.79–1.64) 1.14 (0.70–1.86)	0.77
	Dominant	A/A A/C-C/C	92 (30.7%) 208 (69.3%)	84 (28%) 216 (72%)	1 1.14 (0.80–1.62)	0.47
	Recessive	A/A-A/C C/C	251 (83.7%) 49 (16.3%)	249 (83%) 51 (17%)	1 1.05 (0.68–1.61)	0.83
	Overdominant	A/A-C/C A/C	141 (47%) 159 (53%)	135 (45%) 165 (55%)	1.08 (0.79–1.49)	0.62
DBP (rs7041)	Codominant	G/G T/G T/T	89 (29.7%) 146 (48.7%) 65 (21.7%)	77 (25.7%) 151 (50.3%) 72 (24%)	1 0.84 (0.57–1.22) 0.78 (0.50–1.23)	0.52
	Dominant	G/G T/G-T/T	89 (29.7%) 211 (70.3%)	77 (25.7%) 223 (74.3%)	1 0.82 (0.57–1.17)	0.27
	Recessive	G/G-T/G T/T	235 (78.3%) 65 (21.7%)	228 (76%) 72 (24%)	1 0.88 (0.60–1.28)	0.5
	Overdominant	G/G-T/T T/G	154 (51.3%) 146 (48.7%)	149 (49.7%) 151 (50.3%)	1 0.94 (0.68–1.29)	0.68
DBP (rs4588)	Codominant	C/C A/C A/A	228 (76%) 66 (22%) 6 (2%)	245 (81.9%) 49 (16.4%) 5 (1.7%)	1 1.45 (0.96–2.18) 1.29 (0.39–4.28)	0.2
	Dominant	C/C C/A-A/A	228 (76%) 72 (24%)	245 (81.9%) 54 (18.1%)	1 1.43 (0.96–2.13)	0.074
	Recessive	C/C-C/A A/A	294 (98%) 6 (2%)	294 (98.3%) 5 (1.7%)	1 1.20 (0.36–3.98)	0.76
	Overdominant	C/C-A/A C/A	234 (78%) 66 (22%)	250 (83.6%) 49 (16.4%)	1 1.44 (0.95–2.17)	0.081

*P* < 0.05 was considered statistically significant. Following adjustment for multiple comparisons using the Bonferroni method (0.05/6), *P*-values < 0.0083 were considered statistically significant.

### Haplotype analysis of the CYP2R1 gene

To explore potential associations between CYP2R1 haplotypes and breast cancer risk, a haplotype analysis was performed. The results indicated no significant correlations between the identified haplotypes and disease susceptibility. Detailed findings are presented in Table 5, with no haplotype showing a significant association.

**TABLE 5:** Association between CYP2R1 haplotypes and breast cancer risk.

Gene	Haplotypes		Frequency		Odd ratio (95% CI)	*P*-value
	rs12794714	rs10741657	Control	Cases		
CYP2R1	A	G	0.4914	0.5267	1	–
	G	A	0.2514	0.2201	0.79 (0.59–1.06)	0.12
	G	G	0.2336	0.2133	0.84 (0.63–1.13)	0.25
	A	A	0.0236	0.0399	1.64 (0.76–3.55)	0.21

*P* < 0.05 was considered statistically significant. Following adjustment for multiple comparisons using the Bonferroni method (0.05/6), *P*-values < 0.0083 were considered statistically significant.

### Haplotype analysis of the DBP gene

Haplotype analysis of the DBP gene was conducted to assess potential associations with breast cancer risk. The analysis revealed no significant correlations between the identified haplotypes and disease susceptibility. A summary of these results is provided in Table 6, with none of the haplotypes showing a significant association.

**TABLE 6:** Association between DPB haplotypes and breast cancer risk.

Gene	Haplotypes		Frequency		Odd ratio (95% CI)	*P*-value
	rs7041	rs4588	Controls	Cases		
DPB	G	C	0.5027	0.4741	1	–
	T	C	0.3673	0.4273	0.80 (0.62–1.03)	0.083
	T	A	0.0927	0.0643	1.38 (0.86-2.22)	0.18
	G	A	0.0373	0.0342	1.01 (0.52–1.97)	0.98

*P* < 0.05 was considered statistically significant. Following adjustment for multiple comparisons using the Bonferroni method (0.05/6), *P*-values < 0.0083 were considered statistically significant.

### Association analysis of clinical outcomes and polymorphisms in breast cancer patients

Statistical analyses were utilized to evaluate the relationships between CYP2R1, CYP27B1, CYP24A1, and DBP gene polymorphisms and clinical outcomes in breast cancer patients, as summarized in Table 7. CYP2R1 (rs10741657) was significantly associated with uterine fibroid (*P* = 0.038), distant metastasis (*P* = 0.031), and smoking (*P* = 0.006). CYP27B1 (rs10877012) showed a significant association with estrogen receptor status (*P* = 0.033). DBP (rs7041) was associated with age at first pregnancy (*P* = 0.006) and polycystic ovary syndrome (PCOS) (*P* < 0.00001), whereas DBP (rs4588) was linked to breast cancer stage (*P* = 0.009). Following Bonferroni correction, only the associations of DBP (rs7041) with age at first pregnancy and PCOS and CYP2R1 (rs10741657) with smoking remained significant, while all other associations lost significance.

**TABLE 7:** Genetic polymorphisms and their association with clinical outcomes in breast cancer patients.

Clinical outcome	CYP2R1 (rs12794714)	CYP2R1 (rs10741657)	CYP27B1 (rs10877012)	CYP24A1 (rs2248359)	DPB (rs7041)	DPB (rs4588)
Age	0.447^a^ 0.639	0.557^a^ 0.573	0.029^a^ 0.970	0.755^a^ 0.470	0.061^a^ 0.940	0.539^a^ 0.463
Age at BC Diagnosis	0.344^a^ 0.709	0.899^a^ 0.407	0.007^a^ 0.992	0.443^a^ 0.641	0.070^a^ 0.931	0.409^a^ 0.664
Stage of BC	1.739^a^ 0.181	1.453^a^ 0.239	1.056^a^ 0.349	0.749^a^ 0.475	0.616^a^ 0.541	4.925^a^ 0.009*
Age at first pregnancy	0.234^a^ 0.791	2.062^a^ 0.129	0.017^a^ 0.982	0.132^a^ 0.875	5.105^a^ 0.006*	1.896^a^ 0.152
Body mass index	0.142^a^ 0.867	2.992^a^ 0.051	0.535^a^ 0.586	0.122^a^ 0.884	0.463^a^ 0.629	0.223^a^ 0.800
Age of menarche	0.493^a^ 0.611	0.143^a^ 0.866	1.804^a^ 0.166	0.164^a^ 0.848	0.014^a^ 0.985	1.872^a^ 0.155
Age of menopause	0.915^a^ 0.401	0.109^a^ 0.896	0.367^a^ 0.692	0.040^a^ 0.959	0.537^a^ 0.584	0.220^a^ 0.802
Breastfeeding status	1.786^b^ 0.409	0.322^b^ 0.851	1.206^b^ 0.546	2.753^b^ 0.252	3.549^b^ 0.169	3.240 0.197
Family history of cancer	0.300^b^ 0.860	1.114^b^ 0.572	1.959^b^ 0.375	2.748^b^ 0.253	2.101^b^ 0.349	0.446^b^ 0.800
Other types of cancer	3.197^b^ 0.202	3.479^b^ 0.175	1.291^b^ 0.524	1.682^b^ 0.431	0.465^b^ 0.792	0.676^b^ 0.712
Polycystic ovary syndrome (PCOS)	5.383^b^ 0.067	3.374^b^ 0.185	0.741^b^ 0.690	2.567^b^ 0.277	23.475^b^ < 0.00001*	1.427^b^ 0.489
Uterine fibroid	1.981^b^ 0.371	6.500^b^ 0.038*	0.539^b^ 0.763	2.306^b^ 0.315	0.938^b^ 0.625	0.961^b^ 0.618
Benign breast tumor	3.251^b^ 0.196	1.376^b^ 0.502	1.533^b^ 0.464	5.500^b^ 0.063	1.116^b^ 0.572	0.116^b^ 0.943
Estrogen receptor	0.211^b^ 0.899	0.245^b^ 0.884	6.812^b^ 0.033*	0.162^b^ 0.921	0.650^b^ 0.722	0.037^b^ 0.981
Progesterone receptor	3.060^b^ 0.216	0.703^b^ 0.703	2.909^b^ 0.233	0.040^b^ 0.980	0.281^b^ 0.868	2.912^b^ 0.233
Human epidermal growth factor receptor 2 (HER2)	4.025^b^ 0.133	0.912^b^ 0.633	0.583^b^ 0.747	0.157^b^ 0.924	5.980^b^ 0.050	2.284^b^ 0.319
Axillary lymph node metastasis	0.245^b^ 0.884	0.157^b^ 0.924	3.560^b^ 0.168	1.038^b^ 0.594	1.190^b^ 0.551	5.160^b^ 0.075
Lymph vascular invasion	1.297^b^ 0.522	0.249^b^ 0.882	0.334^b^ 0.846	4.314^b^ 0.115	2.916^b^ 0.232	1.197^b^ 0.549
Distant metastasis	1.098^b^ 0.577	6.931^b^ 0.031*	2.401^b^ 0.300	2.744^b^ 0.253	0.207^b^ 0.901	1.778^b^ 0.410
Allergy	0.866^b^ 0.648	5.344^b^ 0.069	0.513^b^ 0.773	3.708^b^ 0.156	1.088^b^ 0.580	2.175^b^ 0.337
Smoking	0.551^b^ 0.758	9.947^b^ 0.006*	2.761^b^ 0.251	2.032^b^ 0.362	2.870^b^ 0.238	3.107^b^ 0.211

*^a^*F-statistic corresponding to analysis of variance (ANOVA) test.

^b^χ^2^ statistic corresponding to Pearson’s chi-squared test.

*Findings with *p*-values under 0.05 were deemed statistically significant. Following adjustment for multiple comparisons using the Bonferroni method (0.05/6), *P*-values < 0.0083 were considered statistically significant.

### Multivariable binary logistic regression analysis of breast cancer risk predictors

Multivariable logistic regression analysis was performed to evaluate the association between the studied polymorphisms and breast cancer risk after adjusting for potential confounders. Across all models, age, BMI, smoking status, and age at menarche were not significantly associated with breast cancer susceptibility (*p* > 0.05), as presented in Table 8. In contrast, family history of breast cancer consistently showed a strong and significant association with increased risk across all SNP models (OR range: 2.534–2.614, *p* < 0.001).

**TABLE 8:** Binary logistic regression analysis of genetic variants, covariates, and demographic factors associated with breast cancer risk.

SNPs**	Covariate	Odd ratio	Confidence interval 95%	*P*-value*
CYP2R1 (rs12794714)	Age	0.993	0.977–1.009	0.361
	BMI	0.985	0.954–1.017	0.351
	Smoking	0.859	0.528–1.399	0.542
	Age of menarche	0.981	0.869–1.107	0.751
	Family history	2.581	1.787–3.729	0.000*
	A/A	1.647	0.972–2.790	0.064
	A/G	1.629	1.014–2.617	0.044*
	G/G	Reference		
CYP2R1 (rs10741657)	Age	0.993	0.978–1.010	0.423
	BMI	0.985	0.955–1.017	0.354
	Smoking	0.848	0.521–1.380	0.507
	Age of menarche	0.982	0.870–1.107	0.762
	Family history	2.576	1.784–3.718	0.000*
	G/G	1.305	0.658–2.590	0.446
	A/G	1.447	0.717–2.923	0.302
	A/A	Reference		
CYP27B1 (rs10877012)	Age	0.993	0.9771–1.009	0.412
	BMI	0.985	0.955–1.017	0.364
	Smoking	0.845	0.517–1.380	0.501
	Age of menarche	0.979	0.867–1.106	0.735
	Family history	2.534	1.747–3.675	0.000*
	T/T	1.907	0.926–3.928	0.080
	T/G	0.609	0.396–0.939	0.025*
	G/G	Reference		
CYP24A1 (rs2248359)	Age	0.994	0.978–1.010	0.427
	BMI	0.986	0.955–1.017	0.365
	Smoking	0.851	0.524–1.384	0.516
	Age of menarche	0.983	0.871–1.108	0.776
	Family history	2.608	1.808–3.763	0.000*
	A/A	0.858	0.498–1.478	0.582
	A/C	1.063	0.646–1.748	0.810
	C/C	Reference		
DBP (rs7041)	Age	0.993	0.977–1.009	0.393
	BMI	0.985	0.955–1.017	0.357
	Smoking	0.854	0.526–1.388	0.525
	Age of menarche	0.981	0.869–1.106	0.750
	Family history	2.614	1.811–3.773	0.000*
	G/G	0.845	0.508–1.406	0.518
	T/G	1.099	0.698–1.731	0.684
	T/T	Reference		
DBP (rs4588)	Age	0.993	0.977–1.009	0.390
	BMI	0.984	0.953–1.015	0.307
	Smoking	0.937	0.571–1.540	0.798
	Age of menarche	0.986	0.873–1.113	0.816
	Family history	2.608	1.800–3.777	0.000*
	A/A	0.812	0.221–2.975	0.753
	A/C	0.460	0.287–0.739	0.001*
	C/C	Reference		

*P-value < 0.05 consider significant. **The reference category is the control.

Regarding genetic variants, the CYP2R1 rs12794714 polymorphism demonstrated a significant association under the heterozygous model, where the A/G genotype was associated with increased breast cancer risk compared to the G/G reference genotype (OR = 1.629, 95% CI: 1.014–2.617, *p* = 0.044). For CYP27B1 rs10877012, the T/G genotype was significantly associated with a decreased risk of breast cancer (OR = 0.609, 95% CI: 0.396–0.939, *p* = 0.025). In contrast, the DBP rs4588 polymorphism showed a significant association under the heterozygous model, where the A/C genotype was associated with reduced breast cancer risk compared to the C/C genotype (OR = 0.460, 95% CI: 0.287–0.739, *p* = 0.001).

### Overall survival analysis according to genetic variants

**FIGURE 1:**
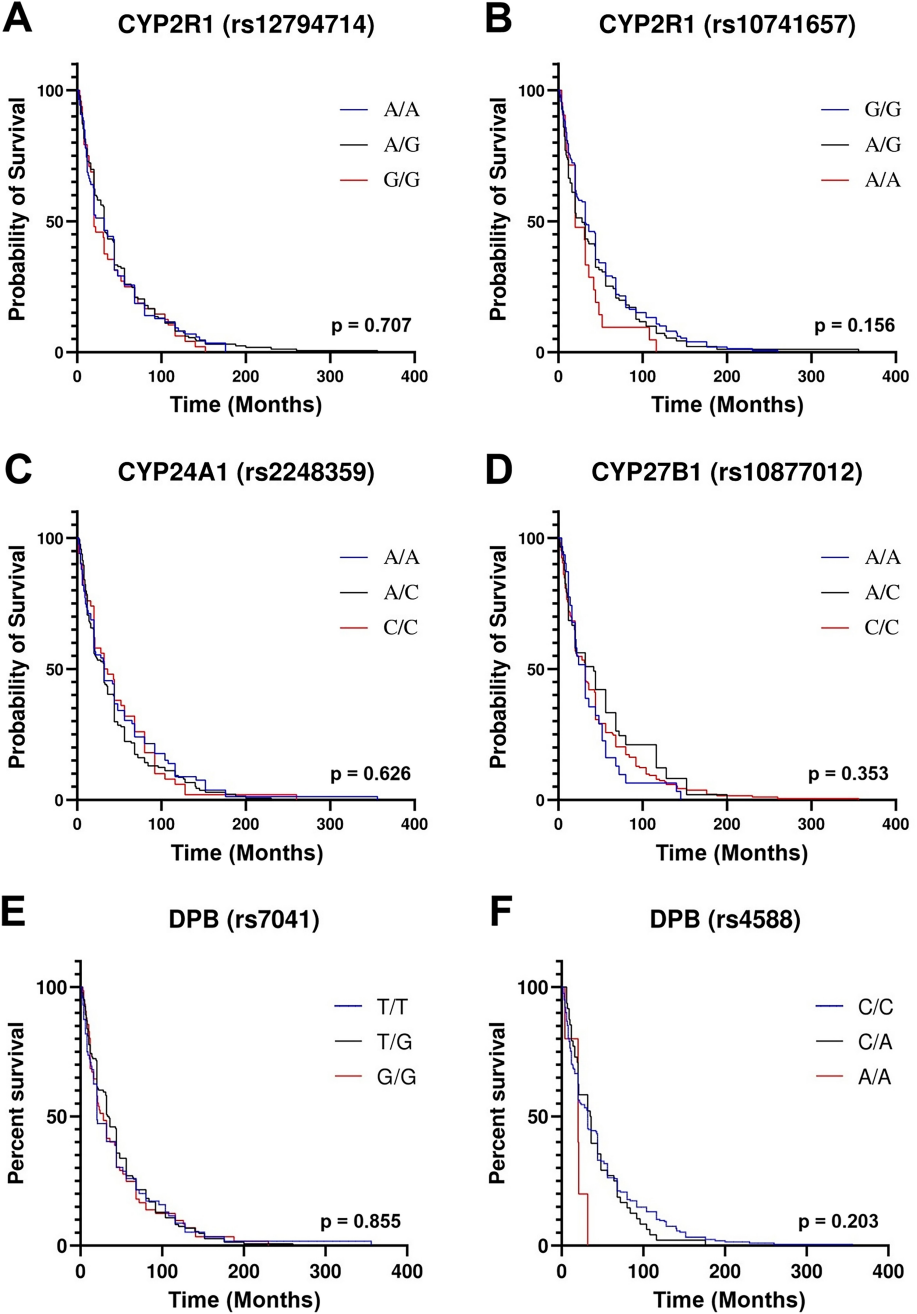
Kaplan–Meier survival curves depicting the association between genotypes and overall survival (OS) for **(A)** rs12794714, **(B)** rs10741657, **(C)** rs2248359, **(D)** rs10877012, **(E)** rs7041, and **(F)** rs4588 polymorphisms. *P*-values were calculated using the log-rank test.

Overall survival was evaluated using Kaplan-Meier survival curves, and the differences were compared using the log-rank test. No statistically significant correlations were observed between survival time and the investigated genetic polymorphisms in CYP2R1 (rs12794714, rs10741657), CYP24A1 (rs2248359), CYP27B1 (rs10877012) or DBP (rs7041, rs4588) among breast cancer patients (*p* = 0.707, 0.156, 0.626, 0.353, 0.855, and 0.203, respectively), as illustrated in Figures 1A–F.

## Discussion

This study investigated the potential association of polymorphisms in key vitamin D pathway genes (CYP2R1, CYP27B1, CYP24A1, and DBP) with breast cancer susceptibility in a Jordanian Arab cohort. Genetic variations in enzymes and binding proteins involved in vitamin D synthesis, activation, degradation, and transport may contribute to cancer susceptibility, including breast cancer. Our findings revealed significant associations for CYP2R1 rs12794714 and CYP27B1 rs10877012, both of which were associated with a lower likelihood of breast cancer, whereas no significant associations were observed for the other polymorphisms examined.

CYP2R1 acts as the primary 25-hydroxylase that converts vitamin D into its circulating form, 25(OH) D, within the liver (45). Genetic polymorphisms in CYP2R1 have been associated with altered 25-hydroxylase activity, thereby affecting circulating 25(OH)D concentrations (46). Serum 25(OH)D concentrations have been implicated in susceptibility to multiple malignancies, including breast (18), gastric (47), thyroid (48), and colorectal cancers (49), among others. Accordingly, increasing research attention has focused on the potential association between CYP2R1 polymorphisms and cancer susceptibility.

In large-scale studies that included the pooled analyses carried out by the Breast and Prostate Cancer Cohort Consortium (BPC3), as well as the Shanghai Breast Cancer Genome-wide Association study, no association was observed in the risk of developing breast cancer in relation to the polymorphism in the CYP2R1 rs10741657 gene (41, 50). Likewise, nested case-control studies and prospective cohort studies investigating vitamin D pathway genes revealed that though CYP2R1 rs10741657 was very strongly associated with levels of 25(OH)D in the blood, it did not have consistent or predictable associations with breast cancer in different populations (51, 52). Consistent with these findings, our study also found no significant association between the rs10741657 polymorphism and breast cancer susceptibility.

The CYP2R1 rs12794714 polymorphism, located in the exon 1 region, is classified as a synonymous variant but may exert functional effects by acting as an exon splicing enhancer (ESE) or silencer (ESS), thereby modulating gene expression (53). The A allele of the rs12794714 variant has been reported to correlate with elevated circulating 25-hydroxyvitamin D levels (14), which may be associated with a lower probability of breast cancer. Limited evidence is present regarding the impact of rs12794714 on breast cancer susceptibility, necessitating further investigation. Evidence from cancer GWAS and case-control studies indicated that the CYP2R1 rs12794714 polymorphism was not significantly associated with breast cancer susceptibility (18, 41). In contrast, our study observed a significant correlation with the GG genotype being linked to a reduced risk of breast cancer.

The CYP27B1 rs10877012 polymorphism has been implicated in breast cancer susceptibility through its interaction with circulating 25(OH)D levels (54). Its effect on cancer risk appears to be influenced by environmental and dietary factors that include UV-B exposure from sunlight and vitamin D intake (18). McGrath et al., in a systematic review, revealed the relationship of this polymorphism to the levels of circulating 25(OH)D3 (55). Regarding the link of the rs10877012 polymorphism with breast cancer risk, several studies have shown no association (18, 41, 56). Contrastingly, the results of the present study reveal that the TG genotype was associated with a reduced risk of breast cancer.

CYP24A1 encodes 24-hydroxylase, which degrades the active vitamin D metabolite 1,25(OH)2D into the water-soluble, biologically inactive compound calcitroic acid (57). This enzymatic pathway is vital for the homeostatic regulation of 1,25(OH)2D levels within the body (58). Consequently, dysregulated expression of CYP24A1 may contribute to disorders related to vitamin D metabolism. Previous studies have reported overexpression of CYP24A1 in breast carcinoma (59). Genome-wide association studies have shown that there are several SNPs in the CYP24A1 gene that are associated with the serum levels of vitamin D (1, 60). Of these, the SNP rs2248359, which is in the promoter region of the gene, seems to regulate the expression of CYP24A1 and has been shown to be associated with several pathological conditions (61–63). Regarding breast cancer, a comprehensive study was carried out by O’Brien et al., who did not find any association between the rs2248359 polymorphism and predisposition to the disease (18), which is consistent with our observation of no association.

Vitamin D-binding protein (DBP) functions as the principal transporter of vitamin D metabolites in circulation, mediating their delivery to target tissues (64). Two non-synonymous variants in the DBP gene, rs7041 and rs4588, have been reported to influence circulating 25(OH)D concentrations (55). Experimental evidence indicates that these two SNPs are associated with modifications in DBP binding affinity (65) and alterations in its metabolic turnover (66). The precise biological mechanism by which alterations in DBP binding affinity and concentration affect circulating 25(OH)D levels is not fully elucidated; however, proposed explanations involve changes in 25(OH)D clearance, renal reabsorption and subsequent metabolism to 1,25-(OH)2D (14, 56, 67). These SNPs are functionally relevant, with rs7041 TT and rs4588 AA genotypes, as well as other DBP variants in linkage disequilibrium, repeatedly associated with reduced circulating 25(OH)D levels in multiple studies, including large-scale GWAS (14, 55, 68).

However, their association with breast cancer remains controversial. Some investigations found no evidence of a relationship between rs7041 or rs4588 and breast cancer susceptibility, as demonstrated in a study of 500 postmenopausal cases and 500 controls from the United States (37). Conversely, Abbas et al. observed a significant protective effect for the combined DBP genotype (rs7041 TT and rs4588 AA) in a German cohort comprising 1,391 postmenopausal cases and 1,365 controls (38). Similarly, one investigation reported a protective effect of the minor alleles of rs7041 and specific combined haplotypes of rs7041 and rs4588 in postmenopausal breast cancer (39, 69). On the contrary, other studies showed that there was no significant correlation (38, 39, 70). In our study, there were no significant associations between the polymorphisms of rs7041 and rs4588 and the susceptibility to breast cancer.

Statistical power analysis was conducted based on genotype frequencies under the most significant genetic models identified for each polymorphism. The CYP2R1 rs12794714 variant showed a significant association with breast cancer susceptibility under the recessive model; however, the corresponding statistical power was moderate (68%), suggesting that this finding should be interpreted with caution and may require validation in larger cohorts. In contrast, the CYP27B1 rs10877012 polymorphism demonstrated a stronger association, particularly under the overdominant model, with adequate statistical power (81%), supporting the robustness and reliability of this result. These findings highlight the importance of aligning power calculations with genotype-based models, as this approach more accurately reflects the underlying genetic architecture and inheritance patterns. Nevertheless, the moderate power observed for some associations underscores the need for further large-scale studies to confirm these findings and to better elucidate the role of these polymorphisms in breast cancer susceptibility.

Hardy-Weinberg equilibrium was assessed for all studied polymorphisms in cases and controls. All variants conformed to HWE expectations, except for the CYP27B1 rs10877012 polymorphism, which deviated significantly (*p* < 0.05) in the case group. HWE testing evaluates the consistency of genotype frequencies with expected distributions in a population under the assumptions of random mating without evolutionary pressures. The deviation may reflect the impact of a small sample size, which can increase genetic drift and affect genotype distribution, as well as other factors such as mutation, selection, gene flow, or non-random mating. The deviation observed in this study may be linked to consanguineous marriages, which are common in the Jordanian population; such non-random mating can decrease genetic diversity and increase the probability of inheriting identical alleles, thereby influencing genotype frequencies and leading to deviations from HWE (71, 72). Evidence from prior research suggests that deviation of specific SNPs from HWE does not necessarily indicate genotyping error but may represent a true association with disease susceptibility. Consequently, removing these variants may lead to the exclusion of relevant biological signals (73–76). Accordingly, the SNP was not excluded solely based on its deviation from HWE, as sensitivity analyses showed consistent results, supporting its inclusion in the analysis.

Although the results obtained in this study have contributed additional information regarding the influence of vitamin D pathway gene variants in breast cancer risk, there are various limitations associated with the study, which are apparent despite the achievement of a moderately sufficient sample size, which may have impeded the study’s sensitivity in relation to the variants’ effect size. The population under investigation was limited to Arab women of Jordanian descent, which might affect the overall generalization of the results to other ethnicities. Environmental and lifestyle factors, such as dietary vitamin D intake and sun exposure, were not comprehensively assessed and could influence the observed associations. The current study’s lack of serum 25(OH)D measurements and functional validation data. Including such measurements would allow direct assessment of vitamin D status in participants, while functional studies could confirm the biological impact of the investigated polymorphisms. Incorporating these data in future research would strengthen the evidence linking genetic variants to breast cancer susceptibility. Future studies addressing these limitations in larger, multi-ethnic cohorts with integrated functional assessments are warranted.

## Conclusion

Our study suggested that CYP2R1 rs12794714 and CYP27B1 rs10877012 be associated with breast cancer susceptibility; the GG genotype of CYP2R1 and the TG genotype of CYP27B1 were associated with lower susceptibility to breast cancer. However, no evident association existed with CYP2R1 rs10741657, CYP24A1 rs2248359, or DBP rs7041 and rs4588 variants. The results of these studies again highlight dissimilar effects of Vitamin D-regulating gene variants in BC risk. Further large studies across different ethnicities are needed for the confirmation of these studies and underlying molecular etiologies.

## Data Availability

The original contributions presented in the study are included in the article/Supplementary material, further inquiries can be directed to the corresponding author.

## Supplementary material

Supplementary Table 1 Detailed genotype data for all analyzed polymorphisms.
